# ﻿A genome survey of *Tetrix japonica* (Insecta, Orthoptera) reveals a comparatively small Tetrigidae genome

**DOI:** 10.3897/zookeys.1254.158678

**Published:** 2025-10-01

**Authors:** Xuejuan Li, Yanpu Chen, Yuxin Liu, Liliang Lin

**Affiliations:** 1 College of Life Sciences, Shaanxi Normal University, Xi’an, China Shaanxi Normal University Xi'an China; 2 Zoological and Botanical Museum, Shaanxi Normal University, Xi’an, China Shaanxi Normal University Xi'an China

**Keywords:** Genome size, high-throughput sequencing, pygmy grasshopper, repetitive sequence

## Abstract

The pygmy grasshopper, *Tetrix japonica*, is a common insect widely distributed in eastern Asia. To further investigate its genome characteristics, high-throughput sequencing was used to obtain genome survey data based on Illumina platforms. Analysis of the results showed that the genome size of *T. japonica* was 1.51 Gb based on K-mer analysis (K-mer = 23), while the size inferred by the flow cytometry (FCM) was 1.94 Gb. The percentage of repetitive sequence was 51.7%, with LINE being the most abundant element (15.46%) and SINE the least abundant element (0.19%). The genome size of *T. japonica* was relatively smaller compared to other orthopteran species, suggesting a fewer repetitive sequences. These genome survey data can be useful for genetic analysis and phylogenetic evolution of *T. japonica* and have potential utility in genomic and biological studies of orthopteran species.

## ﻿Introduction

High-throughput technology has provided a fast and easy method to obtain various types of data, such as mitochondrial genomic, transcriptomic, UCE (ultraconserved element), and whole genomic, and it has played an important role in the development of genomics and evolutionary biology. Many insect genomes have been obtained and used to study the evolution of genome characteristics, such as genome size, gene family composition, and innate immunity. For example, a previous study found that the ancestral insect genome size was 1,069 Mb, that most clades appeared to have undergone massive genome expansions or contractions, and that their genome size variations were the result of selective pressures with a central tendency towards evolutionary optima (Cong et al. 2022). Comparative genome analyses of insects also revealed that gene family expansions or contractions were associated with multiple physiological traits, including the immune system, metabolic detoxification, parasitism, and polyphagy (Li et al. 2019).

With more than 30,304 valid species (Cigliano et al. 2025), Orthoptera (Insecta) (including grasshoppers, crickets, katydids, and their relatives) is the most diverse order among the polyneopteran groups (Song et al. 2015), and locusts and grasshoppers are among the most harmful agricultural pests (Zhang et al. 2019). The species of living Orthoptera belong to one of two monophyletic suborders, Caelifera and Ensifera (Song et al. 2015). To further explore the origin and evolution of orthopteran insects, large-scale genomic data are essential and important, but due to the relatively high repeat sequence and heterozygosity of Orthoptera, the genome assembly data were difficult to obtain. So far, only a few orthopteran insects have had their genomes assembled, such as locusts (Verlinden et al. 2020; Li et al. 2024b) and crickets (Satoh et al. 2021; Ylla et al. 2021; Feng et al. 2022).

For Orthoptera, genome size assessment and repetitive sequence prediction were two important aspects. The K-mer analyses based on genome survey data, flow cytometry (FCM), and whole genome sequencing have been widely used to evaluate the genome sizes of orthopteran species (Mao et al. 2020; Yuan et al. 2021; Sun et al. 2023; Guan et al. 2024; Zhao et al. 2025). The genome sizes of orthoptera varies strongly. For example, genome size measurements of 50 species, including those of Acrididae and Tetrigidae in Caelifera and of Gryllidea and Tettigoniidea in Ensifera, showed genome sizes ranging from 0.95 pg (0.93 Gb) to 2.88 pg (2.82 Gb) in Gryllidea, 2.18 pg (2.13 Gb) to 2.41 pg (2.36 Gb) in Tetrigidae and 1.37 pg (1.34 Gb) to 21.96 pg (21.48 Gb) in the other studied orthopteran species (Hawlitschek et al. 2023). A previous study also showed a significant positive correlation between the proportion of repetitive sequences and genome size in Acrididae species (Zhao et al. 2025). In addition, low-coverage next-generation sequencing data (Nie et al. 2024; Zhao et al. 2025) and whole-genome sequencing (Li et al. 2024b) were used to analyze the repetitive sequence characteristics of orthopteran species.

Tetrigoidea include groups such as pygmy grasshoppers and occupy the relatively basal position of Caelifera (Song et al. 2015, 2020). This group forms a unique family, the Tetrigidae (Cigliano et al. 2025). Previous studies on tetrigid species mostly focused on morphological classification (Long et al. 2023; Bai et al. 2024; Luo et al. 2024), mitochondrial genome sequencing and phylogeny (Lin et al. 2017; Bai et al. 2024; Luo et al. 2024), and transcriptome (Qin et al. 2022; Liu et al. 2023a). The genome sizes of three *Tetrix* species (Tetriginae) were obtained using the FCM method: *Tetrix subulata* (2.22 pg, i.e. 2.17 Gb, from one male individual), *T. tuerki* (2.37 pg, i.e. 2.32 Gb, from two female individuals), *T. undulata* (2.36 pg, i.e. 2.31 Gb, from one female individual and 2.18 pg, i.e. 2.13 Gb from one male individual) (Hawlitschek et al. 2023). The male individuals have a 2n = 12 + X0 chromosome complement (Hawlitschek et al. 2023). In addition, two genome assemblies of tetrigid species have been reported at the chromosome level, including *Eucriotettix oculatus* (Thoradontini) (Li et al. 2024a) and *Zhengitettix transpicula* (Scelimeninae) (Guan et al. 2024), with seven chromosomes identified and Chr5 representing the sex chromosome (X). These high-quality genome data provided available data resources for further study of the origin and evolution of Tetrigidae and even the whole of Orthoptera, but limited genome resources restricted the studies of genome characteristics, origin and evolution, such as the trend of genome size variation.

The pygmy grasshopper *Tetrix japonica* belongs to the Tetriginae, Tetrigidae (Cigliano et al. 2025) and has potential value in genomic and biological studies of Tetrigoidea (Qiu et al. 2017). It is widely distributed in East Asia, and it is distributed in almost all provinces in China except Hainan, where there are no records (Liang and Zheng 1998). It inhabits low grasslands with moss, with the main food being tender moss and humus (Zhang et al. 2023). Most related studies on *T. japonica* have mainly focused on morphological characters (Cao et al. 2015; Pan et al. 2018; Zhang et al. 2023), karyotype (Ma and Zheng 1994), and adaptation (Tsurui et al. 2010). For example, Zhang et al. (2023) examined life cycles and some other biological traits and found that pronotum and wing morphs represent polymorphisms that may be important in evolutionary adaptation. In addition, molecular studies of *T. japonica* have focused on the analysis of mitochondrial genome, nuclear segment and phylogeny (Xiao et al. 2012; Lin et al. 2017), transcriptome sequencing (Qiu et al. 2017), and gut microbiota (Liu et al. 2023b). For example, based on transcriptome data from the Illumina sequencing platform, Qiu et al. (2017) found putative genes involved in pigment pathways, juvenile hormone metabolism, and signaling pathways. However, due to the lack of genomic data, no studies have been conducted on the genomic size, repetitive sequences, and evolutionary features of *T. japonica*.

In this study, genome survey data of *T. japonica* were sequenced using the Illumina platform, and genome size was estimated using both K-mer and FCM methods. Further, genome characteristics of genome size and repetitive sequence content of *T. japonica* were analyzed in combination with other species of Orthoptera. In addition, a transposable element (TE) landscape plot of *T. japonica* was performed. These results provide useful genome data for further study of biological characteristics and wild adaptation of *T. japonica* and will be significant resources to explore genome size and repetitive sequence evolution of the whole of orthopteran insects.

## ﻿Materials and methods

### ﻿Specimen collection, DNA extraction, and genome sequencing

*Tetrix japonica* specimens were collected in 2016 in Xi’an, Shaanxi, China, preserved in 100% ethanol and stored at −20 °C in the Zoological and Botanical Museum, Shaanxi Normal University, China. Specimens of *T. japonica* were examined and identified using Zheng’s (2005) identification keys. Photographs of the specimens are shown in Suppl. material 1.

One *T. japonica* individual (adult, female) was studied, and the hind femurs were used for DNA extraction employing the CTAB method (Doyle and Doyle 1987). Briefly, 1 ml of CTAB extract preheated to 65 °C and 2% protease K (20 µl protease K at a concentration of 20 mg/ml) was added to a centrifuge tube containing the sample. The tube was sufficiently vortexed and mixed and then kept at 65 °C for 30 min. The tube was shaken two or three times to ensure sufficient breaking. The sample was centrifuged at 8,000 rpm for 5 min, and the supernatant was transferred to a new centrifuge tube. Then 800 µl chloroform/isoamyl alcohol (24:1) was added to the tube and the internal solution was completely mixed. The sample was centrifuged at 12,000 rpm for 20 min. The supernatant was retained in a new centrifuge tube and an equal volume of chloroform/isoamyl alcohol was added. The solution was inverted and centrifuged at 12,000 rpm for 20 min. The supernatant was transferred to a new centrifuge tube and 2/3 volume of isopropanol and 1/10 volume of sodium acetate (3 M) were added. The tube was completely mixed and placed at 20 °C for 1 h. The sample was centrifuged at 12,000 rpm for 10 min and the supernatant was discarded. The centrifuge tube was dried at 37 °C for 10 min. Then 50–100 µl of sterile ddH_2_O containing 10 ng/µl RNase was added and the DNA precipitate was dissolved and digested at 37 °C for 1 h.

DNA concentration and quality were measured using Nanodrop and Qubit. The small libraries (six of 270 bp and four of 500 bp) were constructed from fragmented random genomic DNA according to the manufacturer’s instructions (Illumina). Sequencing data were generated using the Illumina HiSeq X Ten sequencing platform with PE = 150.

### ﻿Genome size and repetitive sequences estimation

Raw sequencing data of *T. japonica* were processed by removing adaptor sequences and then trimmed, quality checked and controlled using TRIMMOMATIC 0.38 (Bolger et al. 2014). The genome size of *T. japonica* was estimated based on K-mer analysis methods (k = 23) using JELLYFISH 2.3.0 (Marçais and Kingsford 2011). The K-mer distribution was constructed using GENOMESCOPE 2.0 (Ranallo-Benavidez et al. 2020). In addition, recognition and proportion of repetitive sequences and transposable element (TE) landscape plot were identified using DNAPIPETE 1.4c (Goubert et al. 2015) based on the Orthoptera-TElib library (Liu et al. 2024).

In addition, FCM was used to further measure the genome size of *T. japonica* using *Locusta migratoria* (♂ 1C = 6.2 pg DNA) as an internal reference standard. Heads and hind femurs of two *T. japonica* individuals were used to prepare nuclei, while heads of *L. migratoria* were used as internal standard samples, with three replicates performed. Tissue was minced into small samples, placed in a Dounce Tissue Grinder containing 500 ml cold Galbraith buffer and passed through a fully stroke. A further 500 ml of cold Galbraith buffer was added. The solution was filtered through the filter device and then transferred to a centrifuge tube. The solution was centrifuged at 1,000 g for 5 min and the supernatants were discarded. The remaining precipitates were suspended in 500 µl phosphate-buffered saline and 10 µl RNase was added to the samples. The nuclear solution was stained with 30 µl of propidium iodide for 30 min.

Genome size was measured using a NovoCyte 2040R flow cytometer with a 488 nm laser. Nuclear peaks were obtained using NOVOEXPRESS software and genome size was calculated using the following formula: sample genome size = internal standard genome size × (sample 2C mean peak position/internal standard 2C mean peak position).

### ﻿Mitochondrial genome assembly

Genome survey data were used to assemble the mitochondrial genome in NOVOPLASTY 4.3.1 (Dierckxsens et al. 2017), using *T. japonica* (NC_018543) as a seed and reference sequence. The assembled sequences were annotated in the MITOS WebServer (Bernt et al. 2013), and mitochondrial genome features such as gene position and coding strand were also compared with other Tetriginae mitogenomes (Lin et al. 2017).

## ﻿Results and discussion

### ﻿Genome size

**Figure 1.**
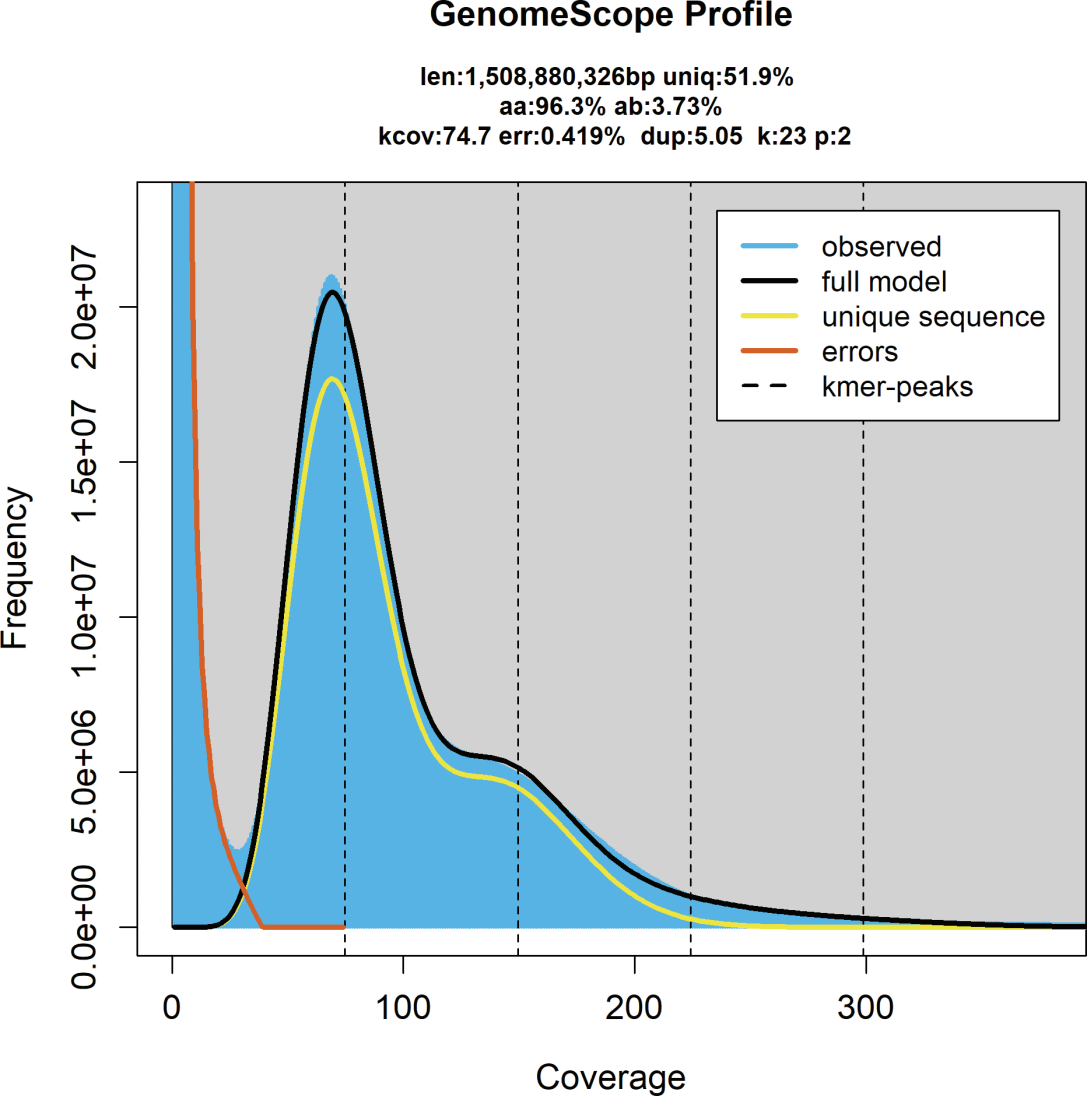
Genome size of *Tetrix japonica* based on K-mer analysis (k = 23).

A total of 350.09 Gb of high-quality sequence data for *T. japonica* was generated from the sequencing libraries with 93.37% Q20, 85.14% Q30 bases and approximately 95.92× coverage (Suppl. material 5: table S1). The genome size of 1.51 Gb was derived from the K-mer analysis (Fig. 1). This genome size was considerably smaller than that of other species of Orthoptera, such as 7.752 Gb of *Neoconocephalus triops* (Hanrahan and Johnston 2021), 13.57–14.34 Gb of eight Pamphagidae species (Nie et al. 2024), 6.45–18.92 Gb of 59 Acrididae species (Zhao et al. 2025), and 2.13–2.32 Gb of three Tetriginae species (Hawlitschek et al. 2023), but larger than two Tetrigidae species including 985.45 Mb in *E. oculatus* (Li et al. 2024a) and 970.40 Mb in *Z. transpicula* (Guan et al. 2024). The heterozygosity was approximately 3.73%, higher than that of 1.12% in *E. oculatus* (Li et al. 2024a), suggesting that *T. japonica* has a relatively complex genome and therefore a relatively high-quality genome assembly may be difficult to obtain.

The FCM analysis provided a high-resolution histogram (Suppl. material 2), and the C-values of the *T. japonica* are shown in Suppl. material 5: table S2. The peak ratio (2.38) meant that the 1C value of *T. japonica* was 1.98 pg (0.32 × 6.2 pg), i.e. 1.94 Gb. This result was closer to the 1.51 Gb derived from the K-mer analysis. The genome size of *T. japonica* estimated by K-mer was smaller than that estimated by FCM, which also occurred in other orthopteran insects such as *Calliptamus abbreviatus* and *Haplotropis brunneriana* (Mao et al. 2020) and other insects (He et al. 2016; Pflug et al. 2020). For example, based on read-depth, K-mer, and FCM methods, Pflug et al. (2020) measured genome sizes in beetles (Coleoptera) and found that all methods tended to underestimate genome sizes. The genome size of one species belonged to the genus *Bembidion* evaluated using most sequence-based methods yielded estimates half that suggested by FCM (Pflug et al. 2020). The discrepancy in genome size estimates between K-mer analysis and FCM may be due to the variability of these two methods or the different samples used for K-mer analysis (He et al. 2016). Additionally, different K-mer values may have generated slight differences in genome size estimates for species. Furthermore, the genome size of female Orthoptera was also significantly larger than that of males. For example, the genome size of females was ~10% larger than males in eight Ensifera species (Mao et al. 2020; Yuan et al. 2021). This difference may be due to the sex chromosome, as most orthopteran insects are XO sexed (Yuan et al. 2021).

### ﻿Repetitive sequence

**Figure 2.**
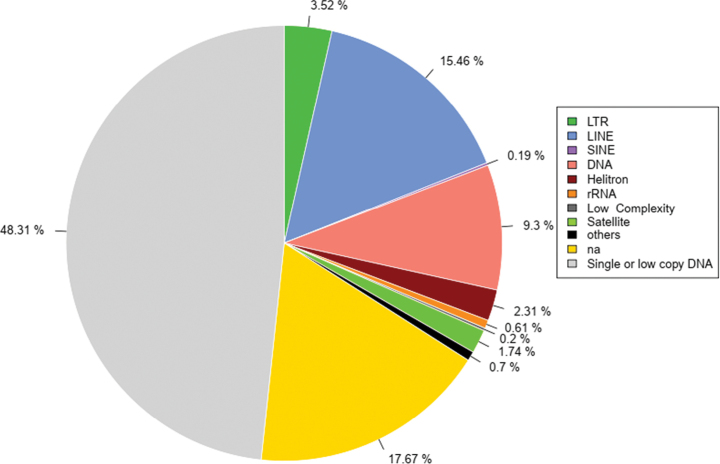
Repetitive sequence proportion of *Tetrix japonica*.

The genome contained approximately 51.7% repetitive sequences in *T. japonica*, which was lower than that of other orthopteran species, such as 57.92–83.58% (Zhao et al. 2025) and 59.9–68.17% (Nie et al. 2024), but higher than that of *E. oculatus* (Thoradontini) of 46.42% (Li et al. 2024a). The two most abundant elements in *T. japonica* were long interspersed nuclear element (LINE) (15.46%) and DNA (9.3%) (Fig. 2), which was different from Acrididae genomes, where the major contributors of repetitive sequences were long terminal repeats (LTR) and LINE (Zhao et al. 2025).

The proportion of LINE in *T. japonica* was higher than in most acridid species, ranging from 6.7% (*Sphingonotus ningsianus*) to 16.33 (*Acrida oxycephala*) (Zhao et al. 2025), but lower than pamphagid species ranging from 19.81% (*Filchnerella nigritibia*) to 24.84% (*Haplotropis brunneriana*) (Nie et al. 2024), and similar to *E. oculatus* (Thoradontini) of 15.61% (Li et al. 2024a). And the proportion of LTR in *T. japonica* was 3.52% (Fig. 2), which was lower than most Acrididae species, ranging from 2.34% (*L. migratoria manilensis*) to 20.98% (*Phlaeoba angustidorsis*) (Zhao et al. 2025), Pamphagidae species ranging from 10.33% (*Pseudotmethis rubimarginis*) to 15.92% (*H. brunneriana*) (Nie et al. 2024). In addition, the proportion of Helitron in *T. japonica* was 2.31%, which was higher than that of Acrididae species ranging from 0% to 0.65% (*Ognevia longipennis*) (Zhao et al. 2025) and Pamphagidae species ranging from 0.15% (*F. rubrimargina*) to 0.31% (*F. nigritibia*) (Nie et al. 2024). Furthermore, the least abundant elements in *T. japonica* were short interspersed nuclear elements (SINE) (0.19%) (Fig. 2), and this proportion was lower than that of Pamphagidae species with ranges from 0.60% (*P. rubimarginis*) to 0.76% (*H. brunneriana*) (Nie et al. 2024). The differences between *T. japonica* and other orthopterans may result in diversity in repetitive sequences in their genomes.

**Figure 3.**
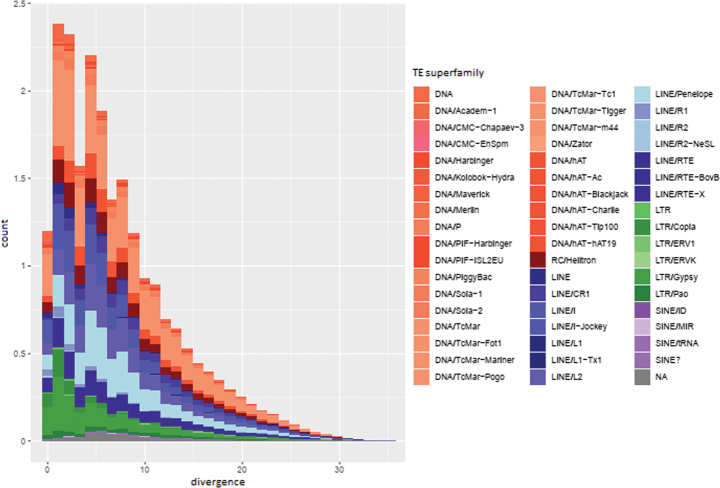
TE landscape plot of *Tetrix japonica*.

LINEs were the most abundant elements in *T. japonica* and may play an important role in the evolution of genome size. Sproul et al. (2023) investigated repetitive elements (REs) in 601 insect species and found that LINEs were also abundant in many insect orders such as Coleoptera, Trichoptera, and Hemiptera. In addition, the TE landscape plot of *T. japonica* showed that there were two transposon burst events, with occurrence times of 2–6 Mya and that DNA/TcMar was the predominant TE element (Fig. 3). The landscape also suggested that TEs had recently undergone active transposition. A previous study showed that TE divergence landscapes exhibited distinct between the acridids *L. migratoria manilensis* and *Angaracris rhodopa* (Liu et al. 2022). Further study of TE divergence, activity, and differences within Tetriginae species is needed using more genome data.

### ﻿Mitochondrial genome

The mitochondrial genome (mitogenome) assembly of *T. japonica* revealed a closed circular DNA molecule of 15,153 bp. The mitogenome contained 37 genes, including 13 protein-coding genes (PCGs), two rRNA genes (rRNAs), and 22 tRNA genes (tRNAs), plus a non-coding control region sequence (A+T-rich region). Of these, four PCGs (ND5, ND4, ND4L, and ND1), two rRNAs (rrnL and rrnS) and eight tRNAs (trnQ, trnC, trnY, trnF, trnH, trnP, trnL1(cun), and trnV) were located in the minor strand (N-strand), while the others were located in the major strand (J-strand) (Suppl. material 3). The mitogenome components of *T. japonica*, such as gene order and coding strand, were consistent with species of Tetriginae (Xiao et al. 2012; Lin et al. 2017).

### ﻿Genome size variation in Orthoptera

The genome size of *T. japonica* was compared to that of other species of Orthoptera in the Animal Genome Size Database (2025-4-3). This database contains the haploid DNA content of 6,534 species, including 3,863 vertebrates and 2,671 invertebrates. Of these, 145 are orthopteran records, representing 10 families (Acrididae, Dericorythidae, Eumasticidae, Gryllacrididae, Gryllidae, Gryllotalpidae, Pamphagidae, Pyrgomorphidae, Tettigoniidae, and Tridactylidae) and 117 species. The genome size of orthopterans in the Animal Genome Size Database ranged from 1.55 pg (*Hadenoecus subterraneus*) to 21.21 pg (*Dericorys annulata*), showing a large variation (~13.68-fold). The genome size of *T. japonica* (1.51 Gb) represented a lower value compared to other species of Orthoptera, and that of Gryllidae was also relatively low (Suppl. material 4).

Grasshoppers contained particularly large variations in genome size, which were also found in the previous study by Schielzeth et al. (2014). For example, based on the ancestral genome size reconstruction results of Ensifera, Yuan et al. (2021) found that the genome size of the grylloid clade tended to decrease, while it expanded substantially in the non-grylloid clades. In addition, a previous study showed that the ancestral genome sizes of Orthoptera, Caelifera, and Ensifera species are 6.19 pg (6.05 Gb), 7.28 pg (7.12 Gb), and 5.37 pg (5.25 Gb), respectively (Hawlitschek et al. 2023). The reasons for the variation in their genome sizes could be as follows. Firstly, the proportion of repetitive sequences was a major factor. Repetitive DNA is the major component of nuclear DNA in most eukaryotic genomes (Biscotti et al. 2015) and can account for up to 90% of genome size (Mehrotra and Goyal 2014). TEs, mobile and repetitive DNA sequences (Teresi et al. 2022), played an important role in the evolution of genome size, structural change, duplication, and functional variability (Majid and Yuan 2021). Several studies focused on insect TEs, mostly based on genome data, and reached many significant conclusions (Boulesteix and Biémont 2005; Petersen et al. 2019; Gilbert et al. 2021; Haq et al. 2022; Liu et al. 2022). For example, in *Arthropoda* species, previous studies have shown a possible relationship between the content and diversity of TEs and genome size (Petersen et al. 2019), and the loads of several TE subfamilies are positively related to genome size (Wu and Lu 2019). Repetitive element expansions, especially of TEs, are also found to be significant drivers of large caddisfly genome sizes (Heckenhauer et al. 2022).

TEs were classified into retrotranposons (class I) and DNA transposons (class II), where class I TEs were moved by a replicative process and amplified by an RNA intermediate, and class II TEs were moved and amplified by DNA (Biscotti et al. 2015; Gilbert et al. 2021). The genome of orthopteran insects contained different proportions of TEs, such as 74.56% TEs of *A. rhodopa*, more than 56.83% in *L. migratoria manilensis* (Liu et al. 2022), and 55% of *Calliptamus abbreviatus* (Majid and Yuan 2021), including some dominant TEs such as LINEs, LTRs, and TcMar-Tc1 (Wu et al. 2017; Majid and Yuan 2021; Liu et al. 2022; Sun et al. 2023). A previous study also showed that the number of TE types detected in a genome increased with genome size, such as DNA transposons of LINEs and LTRs of beetles (Cong et al. 2022). These results suggest that LINEs and LTRs may be the main element of some insects. Cong et al. (2022) analyzed the genomic components of four insect orders and showed that the proliferation of TEs led to high variation in genome size between closely related species. The evolution of larger insect genomes, such as *L. migratoria*, is most likely due to the accumulation of repetitive regions and intron elongation (Wu et al. 2017).

The genome size analyses of orthopterans with respect to repetitive sequences, especially TEs, were important for exploring their genome size evolution. Among Tetriginae, the genome size of *T. japonica* was considerably smaller than in other Orthoptera, suggesting relatively fewer repetitive sequences contained. As Tetrigoidea is considered a primitive group of Caelifera, studying its genome size provides a valuable opportunity to investigate potential mechanisms of genome size evolution in Caelifera and even the whole Orthoptera.

Second, genome expansion and contraction probably also played a role. A previous study showed that genome expansion or contraction events were frequent in insects, resulting in a high diversity of genome sizes (Cong et al. 2022). Third, sexual attractiveness was an important factor in the variation in genome size between males and females. Schielzeth et al. (2014) studied the effect of genome size on sexual attractiveness in *Chorthippus biguttulus* and showed a relationship between song attractiveness and genome size, for example, males with larger genomes had less attractive songs.

## ﻿Conclusions

In this study, a genome survey of *T. japonica* was performed and genome characteristics, such as genome size and repetitive sequence were analyzed in comparison with other species of Orthoptera. The genome size of *T. japonica* was estimated using two strategies: K-mer analysis yielded an estimate of 1.51 Gb, while the FCM method yielded an estimate of 1.94 Gb. In addition, the percentage of repetitive sequences was found to be 51.7%, with LINE and SINE elements being the most and least abundant, respectively. Furthermore, the genome size variation was presented by combining the genome size data of other Orthoptera insects, indicating that *T. japonica* has a relatively smaller genome size.

## ﻿Additional information

### Use of AI

No use of AI was reported.

### Author ORCIDs

Xuejuan Li https://orcid.org/0000-0003-4086-6063

Yanpu Chen https://orcid.org/0009-0001-5037-6570

Yuxin Liu https://orcid.org/0000-0002-0241-1036

Liliang Lin https://orcid.org/0000-0002-9972-4732

### Data availability

The genome survey data have been submitted to the GenBank database under the accession number SRR14460529. The Bioproject and Biosample accession numbers are PRJNA726330 and SAMN18927807, respectively. All data supporting the results of this study are available in the main text or supplementary information.

## Supplementary materials

Supplementary material 1 The specimen of *Tetrix japonica* Data type jpg Explanation note **A** frontal view **B** dorsal view **C** lateral view. https://binary.pensoft.net/file/1428309This dataset is made available under the Open Database License (http://opendatacommons.org/licenses/odbl/1.0/). The Open Database License (ODbL) is a license agreement intended to allow users to freely share, modify, and use this Dataset while maintaining this same freedom for others, provided that the original source and author(s) are credited.Xuejuan Li, Yanpu Chen, Yuxin Liu, Liliang Lin

Supplementary material 2 Flow cytometry result of *Tetrix japonica* Data type png https://binary.pensoft.net/file/1428310This dataset is made available under the Open Database License (http://opendatacommons.org/licenses/odbl/1.0/). The Open Database License (ODbL) is a license agreement intended to allow users to freely share, modify, and use this Dataset while maintaining this same freedom for others, provided that the original source and author(s) are credited.Xuejuan Li, Yanpu Chen, Yuxin Liu, Liliang Lin

Supplementary material 3 Mitochondrial genome of *Tetrix japonica* Data type png Explanation note Mitochondrial genes encoded in the J- and N-strand were shown outside and inside, respectively; tRNA genes are named using amino acid abbreviations. https://binary.pensoft.net/file/1428311This dataset is made available under the Open Database License (http://opendatacommons.org/licenses/odbl/1.0/). The Open Database License (ODbL) is a license agreement intended to allow users to freely share, modify, and use this Dataset while maintaining this same freedom for others, provided that the original source and author(s) are credited.Xuejuan Li, Yanpu Chen, Yuxin Liu, Liliang Lin

Supplementary material 4 Genome size of *Tetrix japonica* combining with 145 records of Orthoptera from the Animal Genome Size Database Data type png https://binary.pensoft.net/file/1428312This dataset is made available under the Open Database License (http://opendatacommons.org/licenses/odbl/1.0/). The Open Database License (ODbL) is a license agreement intended to allow users to freely share, modify, and use this Dataset while maintaining this same freedom for others, provided that the original source and author(s) are credited.Xuejuan Li, Yanpu Chen, Yuxin Liu, Liliang Lin

Supplementary material 5 Additional tables Data type pdf Explanation note **table S1.** Statistics of genome survey sequencing data of *Tetrix japonica*. **table S2.** Genome size of *Tetrix japonica* with *Locusta migratoria* as the internal standard. https://binary.pensoft.net/file/1428313This dataset is made available under the Open Database License (http://opendatacommons.org/licenses/odbl/1.0/). The Open Database License (ODbL) is a license agreement intended to allow users to freely share, modify, and use this Dataset while maintaining this same freedom for others, provided that the original source and author(s) are credited.Xuejuan Li, Yanpu Chen, Yuxin Liu, Liliang Lin
