# Supplementary figures and images for: ﻿A genome survey of Tetrix japonica (Insecta, Orthoptera) reveals a comparatively small Tetrigidae genome
Source: Zookeys. 2025 Oct 1;1254:191–205. doi: 10.3897/zookeys.1254.158678 (PMC12508765; doi:10.3897/zookeys.1254.158678)

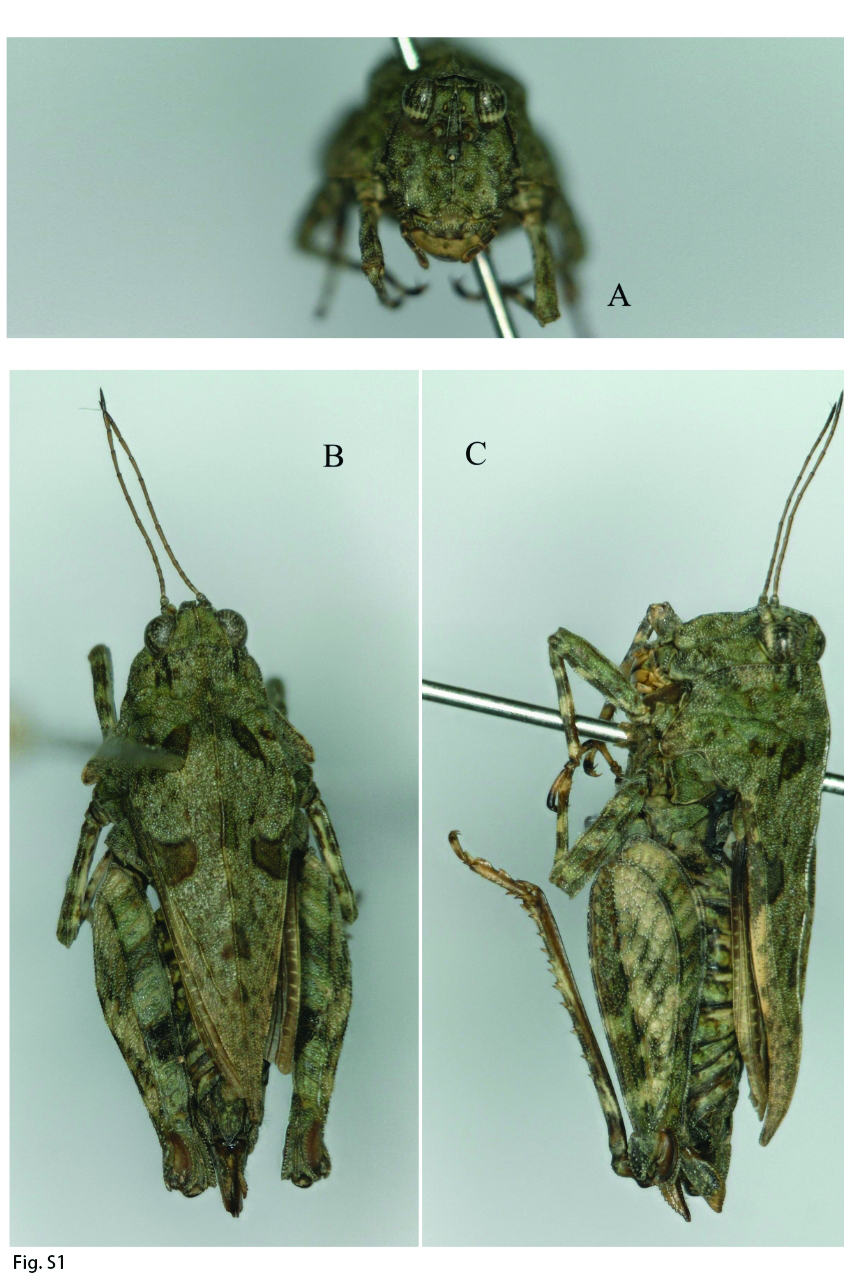

Supplement: Supplementary material 1 — The specimen of Tetrix japonica [file zookeys-1254-191_article-158678__-s001.jpg]

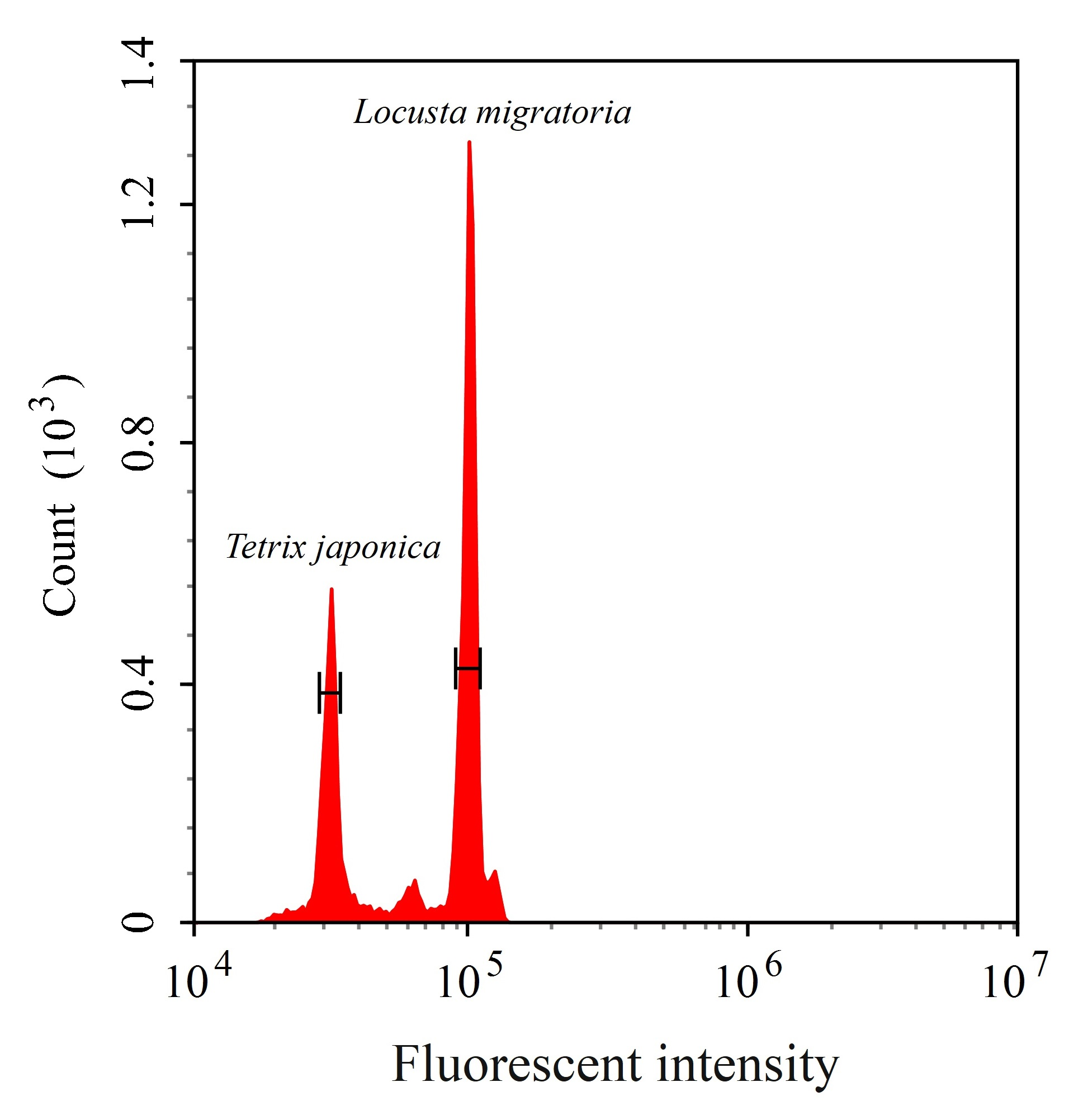

Supplement: Supplementary material 2 — Flow cytometry result of Tetrix japonica [file zookeys-1254-191_article-158678__-s002.png]

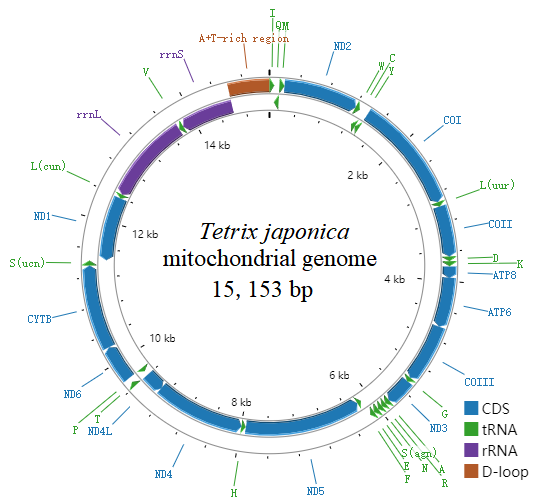

Supplement: Supplementary material 3 — Mitochondrial genome of Tetrix japonica [file zookeys-1254-191_article-158678__-s003.png]

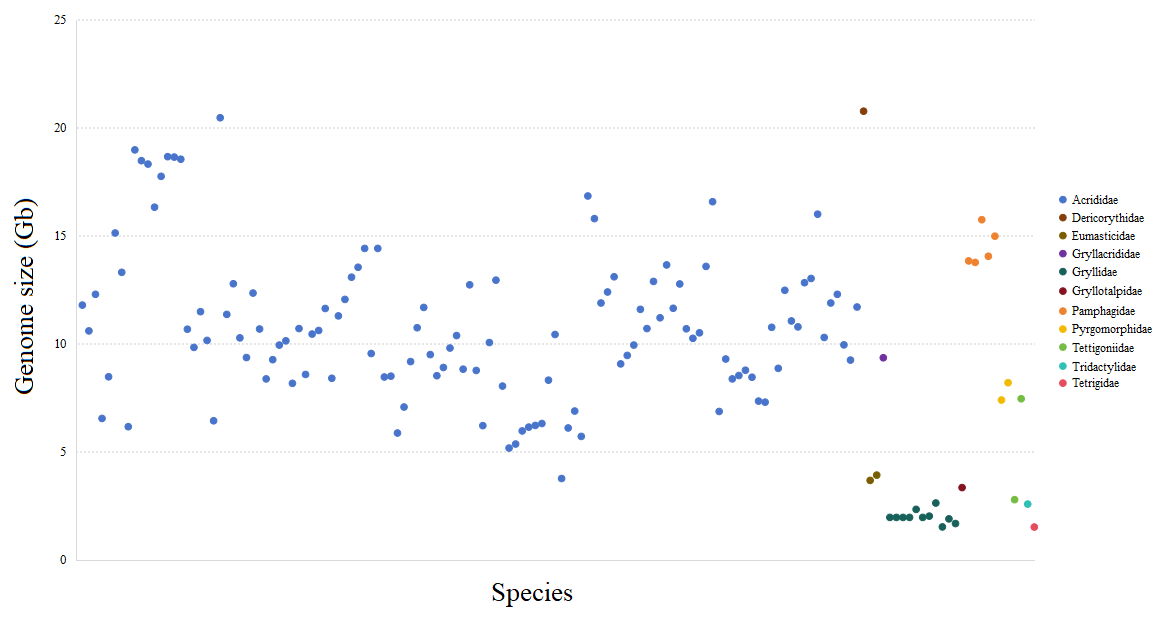

Supplement: Supplementary material 4 — Genome size of Tetrix japonica combining with 145 records of Orthoptera from the Animal Genome Size Database [file zookeys-1254-191_article-158678__-s004.png]
