# Supplementary material for: ﻿A genome survey of Tetrix japonica (Insecta, Orthoptera) reveals a comparatively small Tetrigidae genome
Source: Zookeys. 2025 Oct 1;1254:191–205. doi: 10.3897/zookeys.1254.158678 (PMC12508765; doi:10.3897/zookeys.1254.158678)
Supplement: Supplementary material 5 — Additional tables [file zookeys-1254-191_article-158678__-s005.pdf]

Table S1. Statistics of sequencing data of *Tetrix japonica*.

**Library**	**Data (Gb)**	**Depth (×)**	**Q20 (%)**	**Q30 (%)**
500bp_1	32.99	9.04	94.5	85.31
500bp_2	28.33	7.76	94.51	85.29
500bp_3	30.24	8.29	94.26	85.14
500bp_4	30.88	8.46	94.31	85.16
270bp_1	34.24	9.38	94.44	87.52
270bp_2	37.04	10.15	94.92	88.32
270bp_3	36.46	9.99	94.84	88.21
270bp_4	37.91	10.39	94.72	88
270bp_5	40.41	11.07	93.69	85.81
270bp_6	41.59	11.39	93.37	85.28
Total	350.09	95.92	-	-

Table S2. The genome size of *Tetrix japonica* with *Locusta migratoria* as the internal standard.

**Repeat**	***Locusta migratoria***			***Tetrix japonica***		
	**Mean**	**CV (%)**	**C-value (pg)**	**Mean**	**CV (%)**	**C-value (pg)**
R1	102,974	4.25	6.2	33,191	4.15	2.00
R2	107,305	4.30	6.2	34,105	4.07	1.97
R3	101,658	4.48	6.2	32,238	4.20	1.97
